# Finite Element Analysis of the Mechanical Properties of Axially Compressed Square High-Strength Concrete-Filled Steel Tube Stub Columns Based on a Constitutive Model for High-Strength Materials

**DOI:** 10.3390/ma15124313

**Published:** 2022-06-18

**Authors:** Biao Li, Faxing Ding, Deren Lu, Fei Lyu, Shijian Huang, Zheya Cao, Haibo Wang

**Affiliations:** 1 Hunan Tieyuan Civil Engineering Testing Co., Ltd., Changsha 410075, China; sddonggua@163.com (B.L.); haibarg@163.com (H.W.); 2 China Railway No. 3 Engineering Group Co., Ltd., Taiyuan 030001, China; 3 School of Civil Engineering, Central South University, Changsha 410075, China; dinfaxin@csu.edu.cn (F.D.); lyufei@csu.edu.cn (F.L.); hshijian@cndrealty.com (S.H.); caozheya@csu.edu.cn (Z.C.)

**Keywords:** high-strength concrete-filled square steel tube, finite element analysis, constitutive relation, confinement effect, bearing capacity calculation formula

## Abstract

With the development of new concrete technology, high-strength concrete has been used worldwide. In particular, more economic benefits can be achieved by applying high-strength concrete-filled steel tube (HSCFST) columns in the concrete core walls of super high-rise buildings. A constitutive relation with high applicability for high-strength materials with different strength grades is proposed. Based on this constitutive model, a brick element model of 181 sets of axially compressed square HSCFST members is established and experimentally verified. The effects of the concrete strength, diameter-to-thickness ratio, and steel yield strength on the axial compressive capacities of these members were investigated based on finite element calculation results. The results showed that with an increase in the concrete strength, the ultimate bearing capacities of CS-CC, HS-HC, HS-CC, and CS-HC stub column members increased by 60%, 24%, 44%, and 21% at most, respectively. Additionally, as the steel yield strength increased, the ultimate bearing capacities of CS-CC, HS-HC, HS-CC, and CS-HC stub column members increased by 8.8%, 5.1%, 8.5%, and 5.2%, respectively, Hence, material strength has the greatest impact on CS-CC and HS-CC. The confinement effect of the square steel tube on the concrete weakens as the strength grade of steel or concrete increases. Notably, the confinement effect of steel tube on the concrete is strongest in CS-CC and weakest in the CS-HC. In addition, the confinement coefficients of square HSCFST stub columns with different combinations of concrete and steel strengths were analyzed. Based on the superposition principle in the ultimate state, a practical axial compressive capacity calculation formula for three types of square HSCFSTs is established. Compared with existing major design code formulas, the proposed formula is more accurate and concise and has a clear physical meaning.

## 1. Introduction

Due to their excellent structural properties, concrete-filled steel tube (CFST) columns have been widely applied in super high-rise buildings, urban bridges, long-span bridges, and engineering structures [1,2,3]. Compared with reinforced concrete columns, CFST columns have superior mechanical properties due to the interactions between the steel tube and the concrete. Specifically, in CFST columns, the three-dimensional compression of the concrete due to the confinement by the outer steel tube improves the compressive strength and deformation capacity of the concrete, and the lateral confinement of the concrete by the steel tube reduces the longitudinal stress of the steel tube in the ultimate state. Therefore, CFST columns have better flexural rigidity [4,5], bearing capacity [6], and seismic performance [7]. In addition, CFST columns have better fire [8] and corrosion resistance [9] due to the mutual protective effects of the steel tube and concrete. In practical engineering applications, the use of a CFST structure can significantly reduce the cross-sectional dimension of the columns, thereby reducing the material consumption by more than 50% [10]. CFST structures are easy to manufacture and weld on site and are convenient for concrete pouring with no need for formwork support [11].

In recent years, with the continuous development of materials technology, high-strength materials, such as high-strength steel (HSS), high-strength concrete (HSC), and ultrahigh-strength concrete (UHSC), have been gradually applied to engineering structures. HSS usually refers to steel with a nominal yield stress equal to or greater than 460 MPa [12]. Compared with normal-strength steel, HSS has the advantages of lower construction cost and greater environmental friendliness [13]. In addition, HSS is more suitable for lightweight, high-performance high-rise buildings and long-span structures due to its excellent strength-to-weight ratio, corrosion resistance, and welding performance. However, the application of HSS in practical engineering applications is limited due to the strict restrictions on steel strength grades and imperfect bearing capacity calculation formulas. Range and classification of HSC and UHSC have already been given by Sojobi et al. [14]. HSC has a compressive strength between 50 MPa and 90 MPa, and UHSC has a compressive strength of greater than 90 MPa. Although HSC and UHSC have far greater compressive strengths than normal-strength concrete, their higher brittleness and weaker ductility are not conducive to earthquake resistance and limit their applications in conventional concrete structures [15]. Despite the various restrictions in the application of high-strength materials, UHSC CFST column structures have been applied in the Abeno Harukas building in Osaka, the Star City complex in Sydney, and the Obayashi Technical Research Institute in Tokyo. These UHSC CFST column structures occupied less floor area due to their small cross-sectional areas, which resulted in an increased usable area and more economic benefits. Hence, HSCFST column structures have high research value and promising application prospects.

The steel tube has a lower confinement effect on the concrete, bearing capacity, and ductility in a square CFST column than in a circular CFST column, but square CFST columns are easy to connect and have large cross-sectional moments of inertia, high stability, and good adaptability in terms of earthquake and fire protection measures [16,17]. Researchers have carried out experimental, numerical, and finite element analysis studies on the mechanical properties and practical applications of square HSCFST columns. Yan et al. [18] conducted axial load tests on 32 square UHSC CFST stub columns. Their results showed that the confinement coefficient had a significant effect on the axial load–deformation curves of the specimens and that when the confinement coefficient was between 1.41 and 5.27, the specimens had good ductility. Lai [19] collected 124 sets of axial compression test data of HSCFSTs and compared them with the design method provided by the American Institute of Steel Construction (AISC) specification. Cai and Young [20] experimentally analyzed 26 square CFST stub columns (concrete strength: 34.9 MPa ≤ *f*_c_ ≤ 112.7 MPa, steel strength: 629 MPa ≤ *f*_s_ ≤ 1022 MPa) and conducted extensive numerical analyses on the confinement effect, material, geometry, and contact method. They also evaluated the applicability of the design codes for the compressive strengths of square and rectangular stub columns based on the test results and numerical calculation results. Existing studies have found that the interactions between the steel tube and concrete are related to the strength of the two materials. Therefore, it is necessary to propose formulas for calculating the confinement coefficient and the corresponding bearing capacity that take into account the effect of different material combinations based on the study of the HSCFST stub columns.

In this paper, three types of square HSCFSTs, namely, HSS tubes filled with normal-strength concrete (denoted as HS-CC hereinafter), HSS tubes filled with HSC (denoted as HS-HC hereinafter), and normal-strength steel tubes filled with HSC (denoted as CS-HC hereinafter) are used as the study object. The confinement coefficient of square HSCFST stub columns and a calculation formula for their axial compressive capacity are proposed based on the uniaxial constitutive relation of HSS and concrete proposed by Ding et al. [21] as well as theoretical derivation, numerical simulation, and statistical analysis. The main steps are as follows: A test database (yield strength: 235–960 MPa, compressive strength of concrete: 30–185 MPa) was constructed by collecting the published axial compression test data of square HSCFST stub columns to verify the unified constitutive relation of steel and concrete with different strength grades. The three types of square HSCFSTs with different strength grades were analyzed to find the variation patterns of their confinement effects and the differences in their confinement efficiencies, such as the steel tube yield strength, the concrete strength grade, and the width-to-thickness ratio (steel content). Based on the results of the finite element analysis and static equilibrium theory, the confinement coefficients of the three types of square HSCFSTs were established and the practical bearing capacity calculation formula for the axially compressed square HSCFST stub columns is proposed.

## 2. Introduction to the Constitutive Relation of the Materials

### 2.1. Stress–Strain Relation for Steel

**Figure 1:**
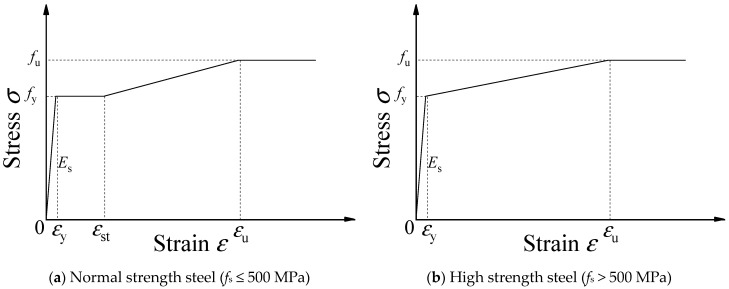
Uniaxial stress–strain curve of steel with or without yield plateau.

The uniaxial tensile stress–strain relation for steel proposed by Ding et al. [22] can be expressed as follows:

(1) $$\sigma =\left\{ \begin{array}{ll} E_{s}\varepsilon & \varepsilon \leq \varepsilon_{y}\\ f_{s} & \varepsilon_{y}<\varepsilon \leq \varepsilon_{\mathrm{st}}\\ f_{s}+E_{\mathrm{st}}(\varepsilon -\varepsilon_{\mathrm{st}}) & \varepsilon_{\mathrm{st}}<\varepsilon \leq \varepsilon_{u}\\ f_{u} & \varepsilon >\varepsilon_{u} \end{array}\right.$$

where *σ* is the stress, *E*_s_ is the elastic modulus (*E*_s_ = 200 GPa), *f*_s_ is the yield strength, *f*_u_ is the ultimate strength, *ε*_y_ is the yield strain, *ε*_st_ is the strain corresponding to the end of the yield plateau, *ε*_u_ is the ultimate strength, and *E*_st_ is the hardening modulus. Then, assuming that the stress of the steel remains constant as the strain increases, the stress–strain relation of the steel is shown in Figure 1. For HSS with no evident yield plateau, *ε*_st_ = *ε*_y_, as shown in Figure 1b.

The literature suggests that the *ε*_st_ of hot-rolled steel with a clear yield plateau should be 0.02 and that the *f*_u_ and *ε*_u_ of hot-rolled steel with different strength grades are expressed as follows:

(2) $$\frac{f_{u}}{235}=0.86\frac{f_{y}}{235}+0.72$$

(3) $$\frac{\varepsilon_{u}}{\varepsilon_{u,235}}=\frac{1}{1+0.15{\left(f_{y}/235-1\right)}^{1.85}}$$

where *ε*_u__,235_ is the ultimate strength of Q235 carbon steel, where Q represents the yield limit of this material, and 235 refers to the yield stress being approximately 235 MPa.

Under axial compression, a CFST column is in a three-dimensional stress state due to the interactions between the steel tube and the core concrete. The equivalent stress *σ_i_* and the equivalent strain *ε_i_* are expressed as follows:

(4) $$\sigma_{i}=\sqrt{\frac{1}{2}[{\left(\sigma_{1}-\sigma_{2}\right)}^{2}+{\left(\sigma_{1}-\sigma_{3}\right)}^{2}+{\left(\sigma_{2}-\sigma_{3}\right)}^{2}]}$$

(5) $$\varepsilon_{i}=\sqrt{\frac{1}{2{\left(1+v_{s}\right)}^{2}}[{\left(\varepsilon_{1}-\varepsilon_{2}\right)}^{2}+{\left(\varepsilon_{1}-\varepsilon_{3}\right)}^{2}+{\left(\varepsilon_{2}-\varepsilon_{3}\right)}^{2}]}$$

where *σ*_1_, *σ*_2_, and *σ*_3_ and *ε*_1_, *ε*_2_, and *ε*_3_ are the principal stresses and strains, respectively, of the outer steel tube and vs. is the Poisson’s ratio of the steel tube, which is defined as follows:

(6) $$v_{s}=\left\{ \begin{array}{ll} 0.285 & \varepsilon_{i}\leq 0.8\varepsilon_{y}\\ 1.075(\sigma_{i}/f_{y}-0.8)+0.285 & 0.8\varepsilon_{y}<\varepsilon_{i}\leq \varepsilon_{y}\\ 0.5 & \varepsilon_{i}>\varepsilon_{y} \end{array}\right.$$

### 2.2. Uniaxial Stress–Strain Curve of the Core Concrete

A unified constitutive relation applicable to concrete with different strength grades has been previously proposed [22]. The stress–strain relation of the core concrete in a CFST column can be expressed as follows:

(7) $$y=\left\{ \begin{array}{ll} \frac{A_{i}x+(B_{i}-1)x^{2}}{1+(A_{i}-2)x+B_{i}x^{2}} & x\leq 1\\ \frac{x}{\alpha_{i}(x-1{)}^{2}+x} & x>1 \end{array}\right.$$

In the formula, the physical meaning of parameter *A_i_* is the ratio of the elastic modulus to the peak secant modulus of the concrete, and *B_i_* is a parameter that controls the degree of attenuation of the elastic modulus of the ascending segment of the stress–strain curve.

When concrete is subjected to uniaxial compression, *y* = *σ*/*f*_c_ and *x* = *ε*/*ε*_c_ in Equation (7), where *σ* is the stress in MPa, *f*_c_ is the axial compressive strength, *f*_c_ = 0.4 *f*_cu_^7/6^, *ε* is the strain, *ε*_c_ is the peak compressive strain, and *ε*_c_ = 420 *f*_cu_^7/18^ × 10^−6^. At this time, *i* = 1, and *A*_1_ and *B*_1_ are the parameters of the ascending segment (*A*_1_ = 6.9 *f*_cu_^−11/30^, *B*_1_ = 1.67(*A*_1_ − 1)^2^), and *α*_1_ is a parameter of the descending segment (*α*_1_ = 4 × 10^−3^ *f*_cu_^1.5^ considering that the parameter of the descending segment is relatively low due to the high brittleness of concrete with a cube compressive strength exceeding 60 MPa).

To achieve the proposed stress–strain relation in the finite element model analysis, the Poisson’s ratio of the core concrete was defined as 0.2. The elastic modulus of concrete with different strengths is expressed as follows:

(8) $$E_{c}=9500f_{\mathrm{cu}}^{1/3}$$

## 3. Finite Element Theoretical Analysis

Due to the limitations of experimental conditions and material properties, the large amount of collected HSCFST test data still cannot cover the parameter matching required for the study. Therefore, it is necessary to use the ABAQUS finite element calculation software for calculation and analysis. The interactions between the steel tube and core concrete can be rationally analyzed by adopting suitable element types and meshing, material properties (constitutive model), steel tube–concrete interface simulation, loading method, and boundary conditions.

### 3.1. Element Type and Meshing

**Figure 2:**
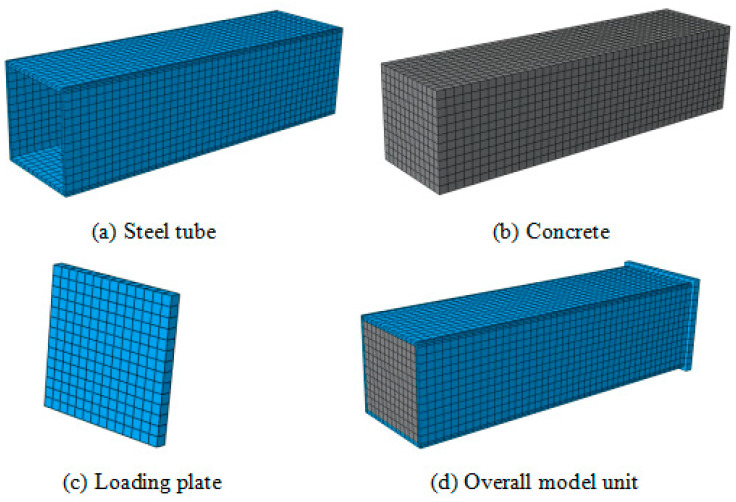
Schematic diagram of model unit.

Both the outer steel tube and the core concrete are analyzed using refined modeling with three-dimensional eight-node brick elements (C3D8R), and each node has three translational degrees of freedom. In the study of mesh convergence, the optimal finite element mesh is determined to provide a relatively accurate solution at a relatively low computation cost. Hassanein [23] noted that mesh refinement has little effect on the numerical results and confirmed that its effect on the *N*–*ε* curve was negligible by testing the mesh convergence of the model with a coarse mesh. Therefore, the axial element size is selected to be 2.5 times the lateral element size. Based on the study of mesh convergence, the cross-sectional element size of the square column is set at *B*/10, where *B* is the width of the square steel tube, as shown in Figure 2.

### 3.2. Material Constitutive Model

The material constitutive model for compressed steel and concrete with different strength grades is proposed based on Section 2. The Poisson’s ratio of the elastic part of concrete under uniaxial compressive stress ranges from 0.15 to 0.22, and the representative values of Poisson’s ratio of concrete are 0.19 to 0.2 in the American Society of Civil Engineers (ASCE) Standards and 0.2 in the National Standards of China. Hence, the Poisson’s ratio (*ν*_c_) of concrete is set to 0.2 in the numerical simulation. The flow potential eccentricity e, the viscosity coefficient *α*, *K*, and *f*_cc_/*f*_c_ are set at 0.1, 0.0005, 2/3, and 2/3, respectively. When the concrete strength is less than 100 MPa, the dilation angle is set to 40°, and when the concrete strength is greater than 100 MPa, the dilation angle is set to 30°. These parameter settings have been widely applied in finite element numerical simulations [24,25,26].

### 3.3. Interface Simulation

The interactions between the steel tube and concrete are usually simulated by the surface-to-surface contact technique. A contact surface pair composed of the inner surface of the steel tube and the outer surface of the core concrete is defined. A hard contact mode is set at the interface in the normal direction. This mode allows the interface to separate after stretching and disallows penetration after compression. The tangential contact can be simulated using the Coulomb friction model, with a friction coefficient of 0.5. There is almost no relative slip between the steel tube and the concrete of a stub column since they bear the load together. A rigid body loading plate with an elastic modulus of 1 × 10^12^ MPa and a Poisson’s ratio of 1 × 10^−7^ is set at the top of the column. The contact between the loading plate and the column is taken as a tie connection, with high stiffness and good convergence. Therefore, the loading surface is taken as the master surface, and the column surface contacting the loading surface is the slave surface.

### 3.4. Boundary Conditions and Loading Methods

**Figure 3:**
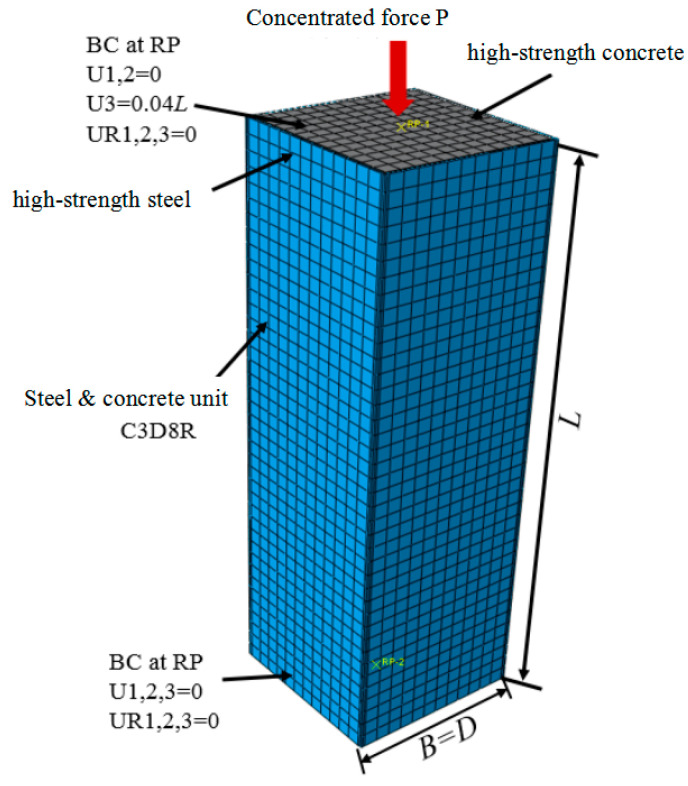
Mesh size selection and schematic view of the FE model.

The bottom surface of the CFST column member has a fixed boundary condition, the top surface of the member has a free boundary, and only the displacement of the loading end in the loading direction is allowed. A static uniform load is proportionally applied in several load increments in displacement control mode on the top of the loading plate using the improved Riks method in the ABAQUS library. Equilibrium iteration is performed at each load increment, and the equilibrium path is tracked in the load–displacement space. This method is a strong nonlinear analysis method often used in static analysis. Nonlinear geometric parameters are introduced to handle large displacement analysis. The finite element model is shown in Figure 3.

### 3.5. Model Validation

**Figure 4:**
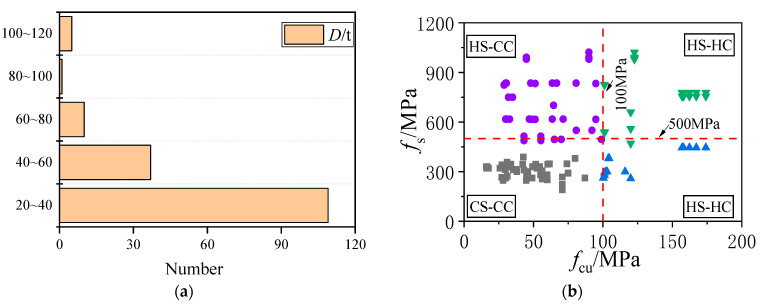
Distribution of test data of CFST stub columns. (**a**) Diameter-to-thickness ratio distribution of steel tube. (**b**) Different material matches distribution.

Figure 4 shows the 181 sets of experimental data samples collected from the literature on CFST columns composed of steel tubes and concrete with different strength grades. The test database does not contain data samples of axially compressed stub columns with excessive steel content, excessively small diameter-to-thickness ratios, or excessively large slenderness ratios. Considering that the materials in the constitutive model used in this study are hot-rolled steel and plain concretes, the test database also does not contain axial compression test data samples of stub columns containing stainless steel, aluminum alloys, or concrete reinforced with other materials (steel fiber, carbon fiber) under axial compression.

It can be observed that the constructed database contains a diameter-to-thickness ratio ranging from 20–120, a steel yield strength ranging from 175–1100 MPa, and a concrete compressive strength ranging from 20–190 MPa, which covers the general interest of engineering practice and academic research. All the published experimental results and relevant HSCFST parameters are shown in Appendix A Table A1.

The strength distribution pattern of the test data points is shown in Figure 4b. Approximately 82% of the test data points are concentrated in concrete strengths of less than 100 MPa, and 66% of the test data points are concentrated in *D*/*t* ratios ∈ [20, 60]. The concrete is categorized into normal-strength concrete (*f*_cu_ < 100 MPa) and HSC (*f*_cu_ ≥ 100 MPa) according to the dilation angle, and the steel is categorized into normal-strength steel (*f*_s_ < 500 MPa) and HSS (*f*_s_ ≥ 500 MPa) according to whether the steel has a yield plateau.

**Figure 5:**
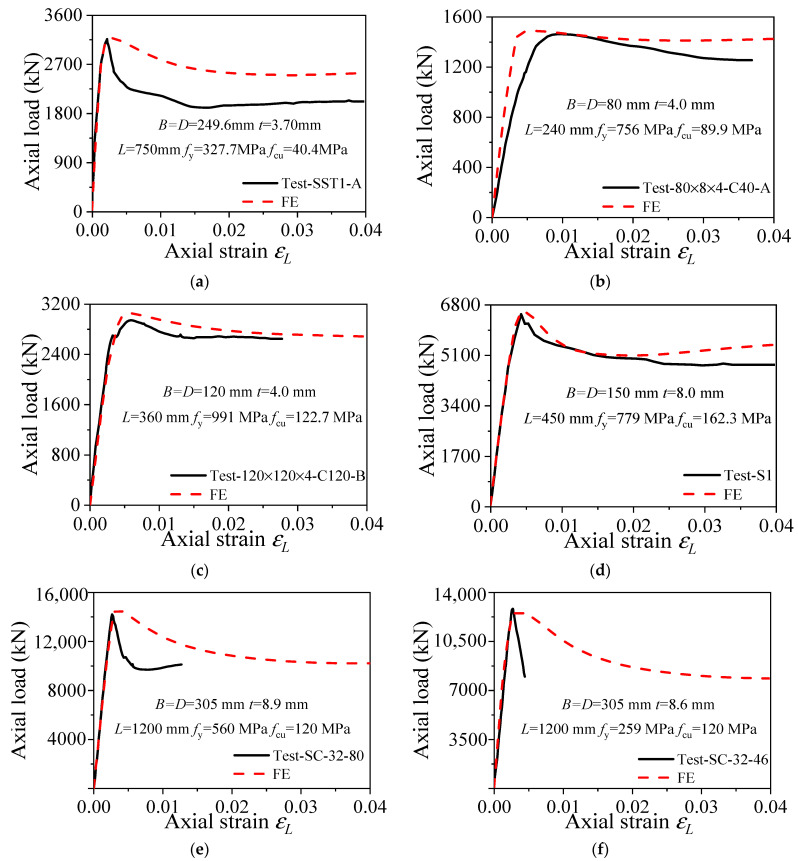
Comparison of FE and experimental load–strain curves of HSCFST. (**a**) Test-SST1-A [27]; (**b**) Test-80804-C40-A [20]; (**c**) Test-1201204-C120-B [20]; (**d**) Test-S1 [28]; (**e**) Test-SC-32-80 [29]; (**f**) Test- SC-32-46 [29].

The finite element modeling method used in this paper is applied to compare and analyze the axial compression test results of the square HSCFST stub columns provided in the literature. The verification of the typical finite element model is shown in Figure 5, which compares the finite element simulation curve of a typical square CFST stub column under axial compression with the experimental results in the literature. The comparison of the analysis results in Appendix A Table A1 shows that the ratio (*N*_u,e_/*N*_u,FE_) of the measured ultimate bearing capacity (*N*_u,e_) to the ultimate bearing capacity calculated based on the finite element model (*N*_u,FE_) is 1.02, with a dispersion coefficient of 0.038. The *N*_u,FE_ values are generally in good agreement with *N*_u,e_, especially in the elastic stage, during which the measured and calculated curves basically overlap. Figure 5 compares the finite element simulation curves of typical square HSCFST stub columns under axial compression and the experimental curves obtained from the literature. Hence, the bearing capacity–strain curves calculated based on the finite element model are generally in good agreement with the experimental curves obtained from the literature. There are obvious differences in the descending section of the test curve of some specimens, as shown in Figure 5a,e,f. Due to the influence of various factors in the experiment, the material strength cannot be fully exerted. After reaching the limit state, local defects may appear between the steel tube and concrete, manifesting as a rapid decrease in bearing capacity and poor ductility. Since only experimental data can be collected from the literature, and the specific conditions of the specimen and experimental status can only be known from pictures, so the influence of various defects is not considered in the modeling, and there are differences between some test curves and the descending section of the finite element calculation curve. After comprehensive consideration, this modeling method is reasonable and reliable.

### 3.6. Parameter Analysis

In the finite element model, the width (*B* = *D*), length (*L*), and wall thickness (*t*) of the square HSS tube are set to 500 mm, 1500 mm, and 3, 6, or 10 mm, respectively. The steel content (*ρ*_s_) is between 0.02 and 0.08, the yield strength of the steel (*f*_s_) is 235, 345, 460, 550, 690, 780, or 960 MPa, and the cube compressive strength of the concrete (*f*_cu_) is 40, 70, 100, 120, 150, or 180 MPa. To explore the optimal combination of concrete strength and tube strength, these concrete strength and steel yield strength values were paired up to form a total of 126 model groups, and the model parameters are shown in Table 1. In this paper, HSS tubes filled with HSC were denoted as HS-HC, HSS tubes filled with normal-strength concrete were denoted as HS-CC, normal-strength steel tubes filled with HSC were denoted as CS-HC, and normal-strength steel tubes filled with normal-strength concrete were denoted as CS-CC. The three types of HSCFSTs, namely, HS-HC, HS-CC, and CS-HC, are the focus of analysis in this paper.

**Figure 6:**
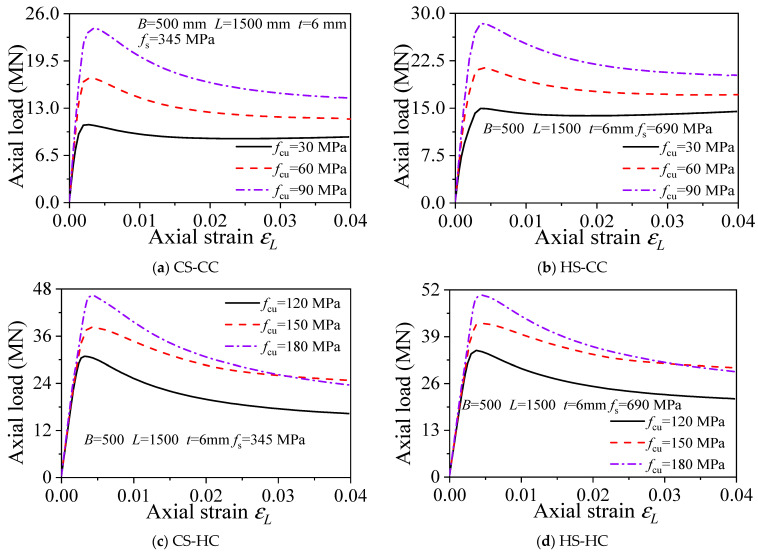
Influence of concrete strength on the load–strain curve.

**Figure 7:**
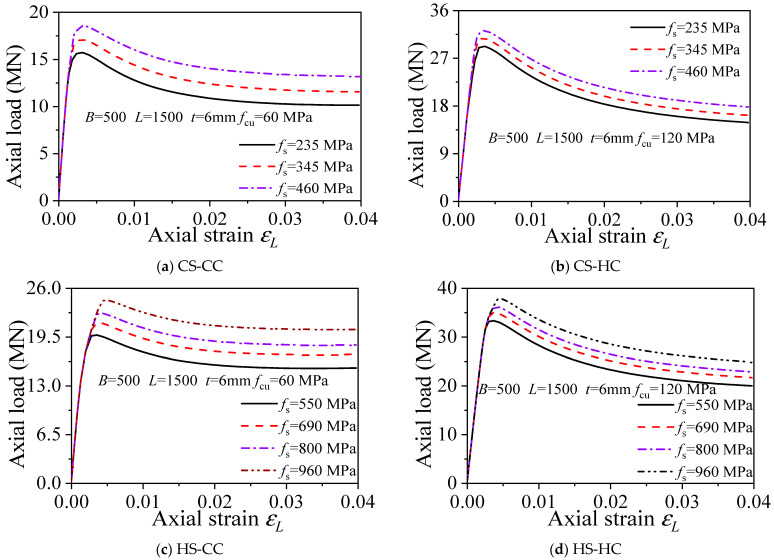
Influence of steel yield strength on the load–strain curve.

**Figure 8:**
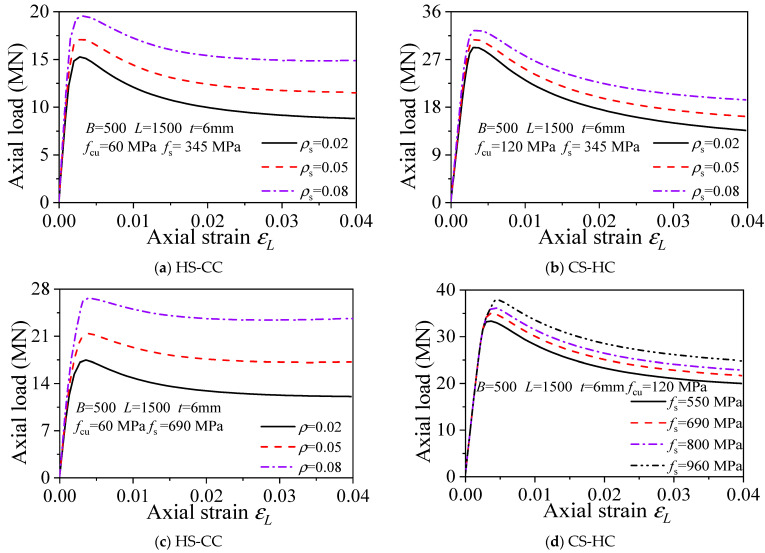
This is a figure. Schemes follow the same formatting.

The effect of concrete strength on the mechanical properties of square HSCFST stub columns was analyzed using a steel tube wall thickness (*t*) of 6 mm and steel yield strengths (*f*_s_) of 345, 460, 690, and 960 MPa. Figure 6, Figure 7 and Figure 8 show the effect of different parameters on the axial compressive capacity of the square HSCFST stub columns. Specifically, the concrete strength grade, the steel yield strength, and the steel content all have a certain effect on the load–displacement curve and the bearing capacity.

#### 3.6.1. Concrete Strength *f*_cu_

Figure 6 shows the load–longitudinal strain (*N*–*L*) curves of axially compressed square HSCFST columns with different *f*_cu_ values. The effects of concrete strength on the mechanical properties of axially compressed square HS-HC, HS-CC, CS-HC, and CS-CC stub column members with a tube wall thickness (*t*) of 6 mm and concrete strengths (*f*_cu_) of 30, 60, 90, 120, 150, and 180 MPa were analyzed. The results show the following: (1) Under the premise that other parameters remain constant, the concrete strength *f*_cu_ is one of the factors influencing the bearing capacity of the CFST members. The initial stiffness of the members is little affected by *f*_cu_, and the ductility increases with increasing *f*_cu_. (2) With an increase in the concrete strength, the ultimate bearing capacities of CS-CC, HS-HC, HS-CC, and CS-HC stub column members increased by 60%, 24%, 44%, and 21% at most, respectively. Hence, the concrete strength has the greatest impact on CS-CC, the second greatest impact on HS-CC, and the least impact on CS-HC.

#### 3.6.2. Steel Yield Strength *f*_s_

The effects of steel yield strength (*f*_s_) on the load–displacement curves of axially compressed square HS-HC, HS-CC, CS-HC, and CS-CC stub column members with a tube wall thickness (*t*) of 6 mm and steel yield strengths (*f*_s_) of 235, 345, 460, 550, 690, 800, and 960 MPa were analyzed, as shown in Figure 7. The analysis results show the following: (1) Under the premise that other parameters remain constant, the initial stiffness of the HS-HC, HS-CC, CS-HC, and CS-CC stub column members is almost the same, and the steel yield strength has almost no effect on the stiffness of the members. (2) The ultimate bearing capacity increases with increasing steel yield strength *f*_s_, which indicates that the steel yield strength is one of the factors influencing the bearing capacity of the CFST members. As the steel yield strength increased, the ultimate bearing capacities of CS-CC, HS-HC, HS-CC, and CS-HC stub column members increased by 8.8%, 5.1%, 8.5%, and 5.2%, respectively. Hence, steel yield strength has the greatest impact on CS-CC and HS-CC, the second greatest impact on HS-HC, and the least impact on CS-HC.

#### 3.6.3. Width-to-Thickness Ratio (B/t)

Figure 8a–d shows the load–displacement curves of the axially compressed square CFST columns with different width-to-thickness ratios. The analysis results of the square HS-HC, HS-CC, CS-HC, and CS-CC stub columns with *B* = 500 mm and *t* = 6 mm show the following: (1) Under the premise that other parameters remain constant, the ultimate bearing capacities of the members increase as the width-to-thickness ratio decreases. When the thickness *t* decreased from 5 mm to 3 mm, the bearing capacities of HS-HC, HS-CC, CS-HC, and CS-CC stub columns increased by 14%, 24%, 5%, and 13%, respectively. Hence, the width-to-thickness ratio (*D*/*t*) has the greatest impact on HS-CC and the least impact on CS-HC. (2) Compared with concrete strength and the steel yield strength, the width-to-thickness ratio has a greater impact on the initial stiffness of the members. The greater the width-to-thickness ratio of a member is, the greater the initial stiffness of the member.

In summary, the influence of material strength and width-thickness ratio on bearing performance is complex. In order to explore the best configuration, it is recommended to use the linear weighting and optimization method proposed by Sojobi [30], which is a simplified multi-criteria decision-making optimization method which can be utilized to select the best structural configuration when several configurations are considered alongside several mechanical properties.

### 3.7. Analysis of the Confinement Effect

**Figure 9:**
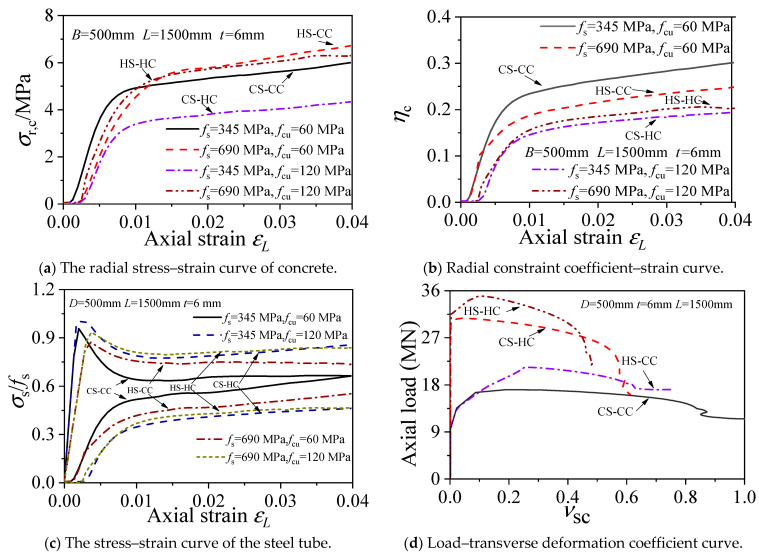
Comparison of axial compressive properties between high-strength specimen and ordinary specimen.

The variation pattern of the longitudinal stress–strain curves and circumferential stress–strain curves of the steel tube used by Ding [11] can reflect the confinement effect of the steel tube on the concrete in a square CFST stub column under axial compression. The confinement effect of the steel tube on the core concrete can be assessed through the equivalent radial stress of the concrete under lateral compression: *σ_r_*_,c_ = 2*tσ_θ_*_,s_/(*B* − 2*t*). The confinement efficiency of the steel tube on the core concrete can be evaluated by the radial confinement coefficient and the time at which the longitudinal stress–strain curve of the steel tube intersects its circumferential stress–strain curve. Figure 9 compares the axial compressive stress and radial confinement coefficient of the square HSCFST stub columns and the common-strength CFST stub columns. As shown in the figure, (1) as the steel strength grade increases, the radial stress of the core concrete (*σ_r_*_,c_) decreases during the early loading stage but increases during the late loading stage, whereas the radial confinement coefficient (*η*_c_) decreases constantly. (2) As the concrete strength grade increases, the *σ_r_*_,c_ always decreases, whereas *η*_c_ decreases in the early loading stage but increases at the late loading stage. (3) During the loading process, the longitudinal stress of the steel tube gradually decreases after reaching the maximum point, while the circumferential stress of the steel tube gradually increases after reaching the minimum point. The intersection point of the longitudinal stress–strain curve and the circumferential stress–strain curve of CS-CC appears first, while that of CS-HC appears last because the HSC has not reached the peak stress when the common-strength steel buckles. Hence, CS-HC has the weakest confinement effect.

## 4. Calculation Formula for Bearing Capacity

### 4.1. Model Simplification and Formula Establishment

**Figure 10:**
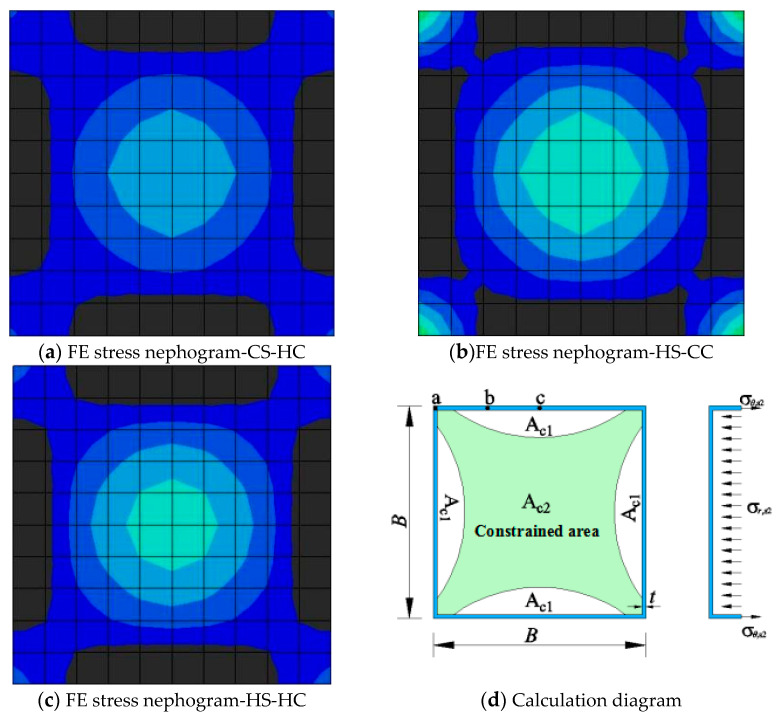
Simplified stress distribution model at the mid-height section of CFST stub columns.

The model is simplified using the superposition principle and stress distribution analysis. *A*_c_ is the cross-sectional area of the concrete, *A*_c__1_ is the unconfined area of the concrete, *A*_c__2_ is the confined area of the concrete, *B* is the cross-sectional length or width of the steel tube of each strength grade, and *t* is the wall thickness of the steel tube of each strength grade. According to the stress distributions in Figure 10a–c, when the core concrete reaches the ultimate state, the relations between *A*_c_, *A*_c__1_, and *A*_c__2_ are expressed as follows:

*A*_c1_ = 0.25*A*_c_ (9)

*A*_c2_ = 0.75*A*_c_ (10)

**Figure 11:**
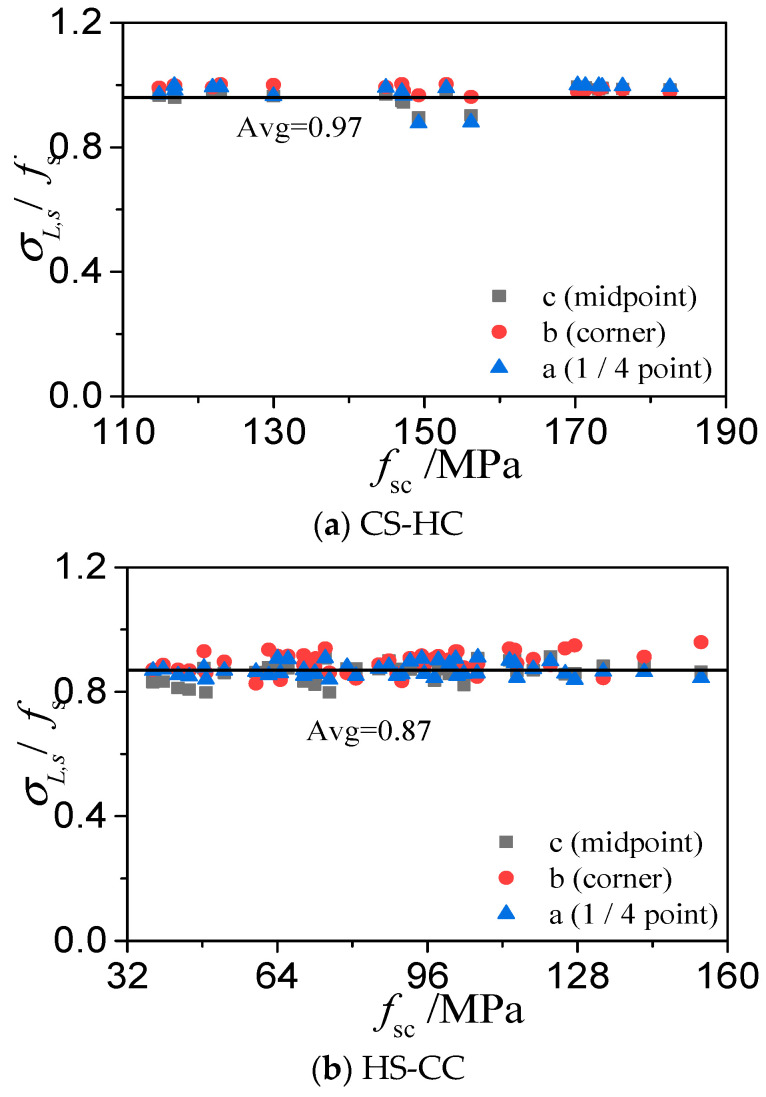

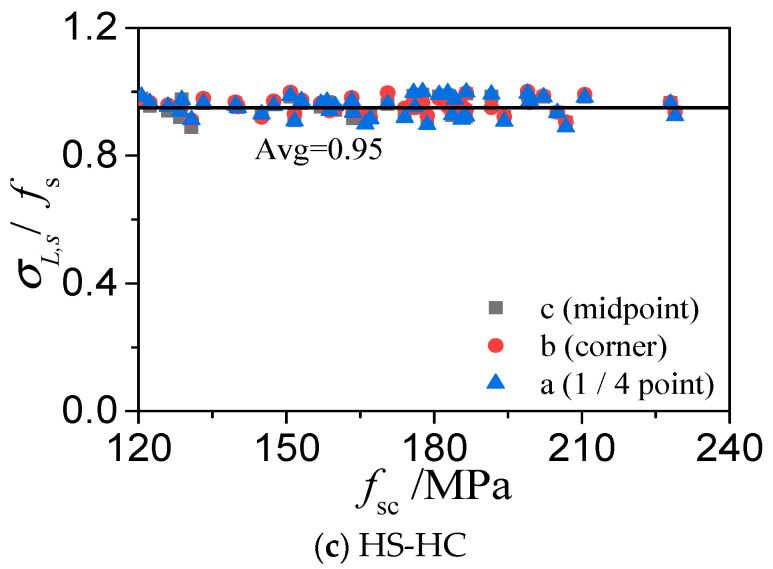
Average ratio of longitudinal stress to yield strength of a high-strength steel tube.

**Figure 12:**
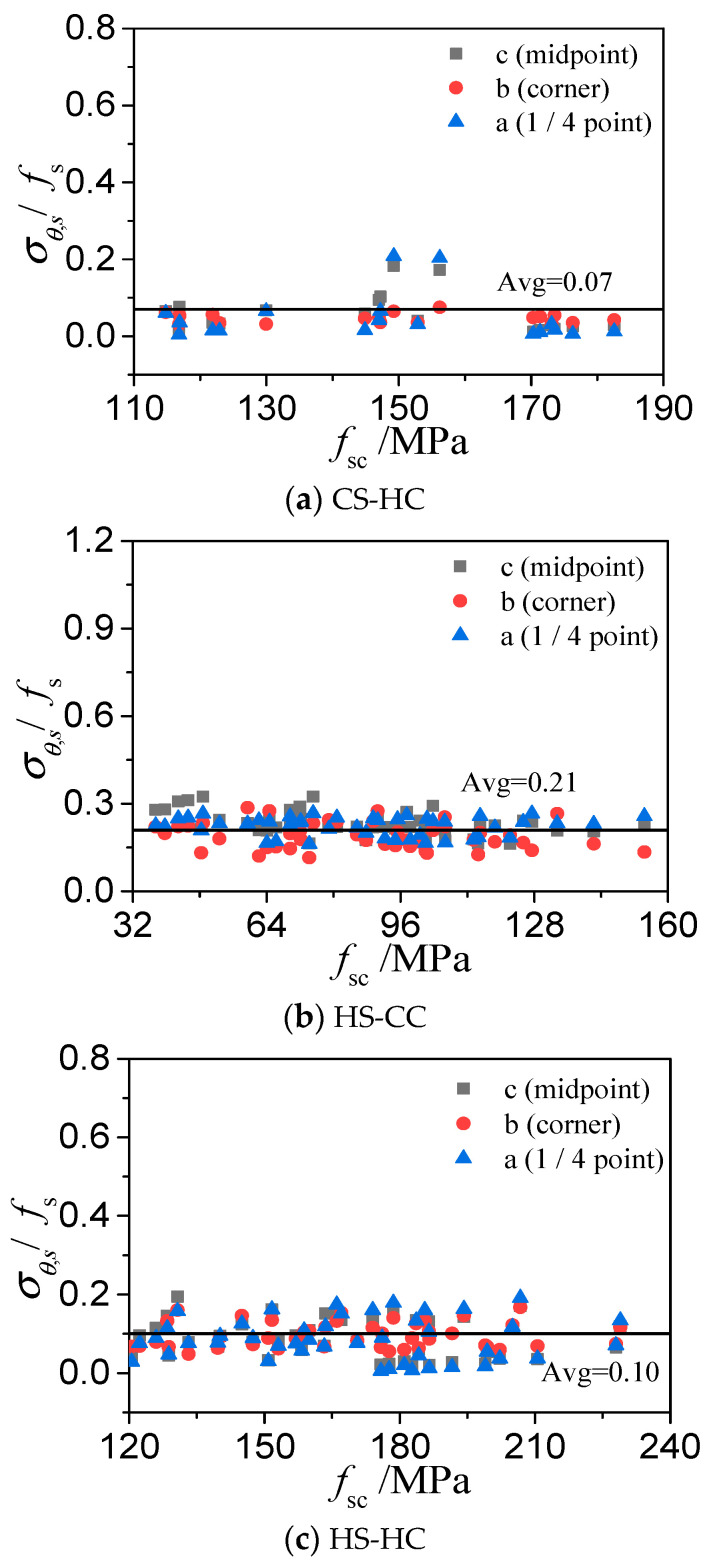
Average ratio of circumferential stress to yield strength of a high-strength steel tube.

Figure 11 and Figure 12 show three points on the cross-sections of the steel tubes at the ultimate bearing capacities of the three types of HSCFST columns: the middle point b, the corner point a, and the 1/4 point c on the same side of the square. The ratio of the longitudinal stress (*σ_L_*_,s_) to the nominal yield strength (*f*_s_) of the steel tube and the ratio of the circumferential stress (*σ_θ_*_,s_) to the fs of the steel tube at these three points varies with the ultimate strength of the specimen (*f*_sc_ = *N*_u_/*A*_sc_, *A*_sc_ = *A*_c_ + *A*_s_), as shown in the figures. When a square HSCFST stub column reaches its axial compressive capacity, the relation between the axial stress and the yield stress and the relation between the circumferential stress and the yield stress of the steel tube are as follows:

*σ_L_*_,*s*_ = *αf*_s_ (11)

*σ_θ_*_,*s*_ = *βf*_s_ (12)

The relation between the radial stress of the core concrete and the circumferential stress of the steel tube shown in Figure 10 is as follows:

(13) $$\sigma_{r,c}=\frac{2t\sigma_{\theta ,s}}{B}$$

The relation between the axial compressive strength *f_L_*_,c_ and the lateral stress *σ_r_*_,c_ of the core concrete in the reinforced zone is as follows:

*f_L_*_,*c*_ = *f*_c_ + 3.4*σ_r,_*_c_ (14)

Based on the static equilibrium condition of the cross-section, the following equation can be obtained:

*N*_u_ = *σ_L_*_,c_*A*_c2_ + *f_L_*_,c_*A*_c1_ + *σ_L_***_,_***_s_A*_s_ (15)

According to Equations (11)–(15), the axial compressive capacity of the square HSCFST (*N*_u_) can be expressed as follows:

*N*_u_ = *f*_c_*A*_c_ + *Kσ_s_A*_s_ (16)

where *K* is the confinement coefficient of the square steel tube to the concrete. Table 2 shows the *K* values of the three types of square HSCFSTs and the *K* values of the CS-CC obtained from the literature. The CS-CC has the best confinement effect among the four types of CFSTs. Among the three types of square HSCFSTs, HS-CC has the best confinement effect, HS-HC has the second-best confinement effect, and CS-HC has the weakest confinement effect.

### 4.2. Formula Verification

**Figure 13:**
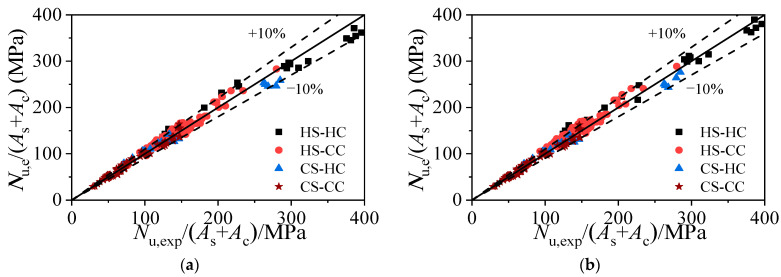
Comparison of experimental results with Equation (16) and FE, respectively. (**a**) Comparison of average ultimate stress from test and Equation (16). (**b**) Comparison of average ultimate stress from test and FE.

Figure 13 compares the axial compressive capacities of the three types of square HSCFSTs calculated using the axial compressive capacity calculation formula and the experimental results of previous studies. In Figure 1, the average stress *N*/(*A*_s_ + *A*_c_) is compared due to the large differences in the cross-sectional sizes of square CFST stub columns used in different axial compression tests. Table 2 shows the statistical results of the comparison between the measured bearing capacities and the bearing capacities calculated using Equation (16) or the finite element model. The bearing capacities calculated using Equation (16) or the finite element model are in good agreement with the measured bearing capacities, although the dispersion coefficient of Equation (16) is slightly larger.

**Figure 14:**
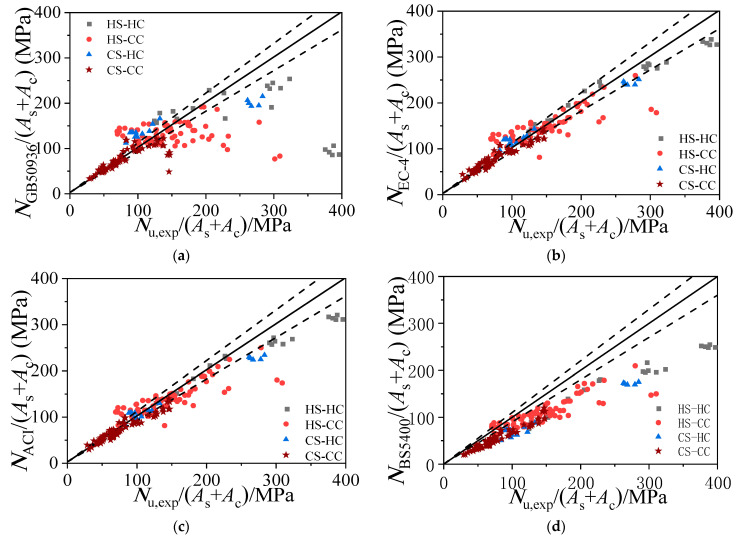
This is a figure. Schemes follow the same formatting. (**a**) Comparison of ultimate bearing stress obtained from test results and GB50936. (**b**) Comparison of ultimate bearing stress obtained from test results and EC4. (**c**) Comparison of ultimate bearing stress obtained from test results and ACI. (**d**) Comparison of ultimate bearing stress obtained from test results and BS5400.

At present, Eurocode 4 (EC4)-2004 [31], the British standard BS5400 [32], the Chinese standard GB50936-2014 [33], and American ACI standards [34] are the major design codes for calculating the axial compressive capacity of CFST. The applicability of the ultimate axial compressive capacities calculated by the formulas recommended in these design codes is analyzed in this paper using a bearing capacity database consisting of the experimental data from the 181 sets of CFST stub columns established in the previous section, and the relevant statistical results are shown in Table 3 and Figure 14. The average ratio of the *N*_u,exp_ to the Nu calculated based on the EC4-2004 is 0.99, with a dispersion coefficient of 0.172. The average ratio of *N*_u,exp_ to the *N*_u_ calculated based on the BS5400 is 1.39, with a dispersion coefficient of 0.142. The average ratio of *N*_u,exp_ to the *N*_u_ calculated based on the ACI standard is 1.07, with a dispersion coefficient of 0.160. The average ratio of *N*_u,exp_ to the *N*_u_ calculated based on the GB50936-2014 is 1.17, with a dispersion coefficient of 0.552. The *N*_u_ calculated based on Equation (16) is in best agreement with the *N*_u,exp_.

## 5. Conclusions

(1) A refined 3D finite element model consisting of 181 sets of axially compressed square HSCFST members is established using the unified constitutive relation of steel and concrete.
(2) A total of 126 groups of examples were constructed to analyze the effects of the diameter-to-thickness ratio, concrete strength *f*_cu_, and steel strength *f*_s_ on the bearing capacity and confinement effect of the members. Increasing the steel yield strength and reducing the concrete strength will weaken the confinement efficiency of the steel tube to the concrete. Among the four types of CFSTs, CS-CC has the strongest confinement effect, while CS-HC has the weakest confinement effect. Compared with concrete strength and the steel yield strength, the width-to-thickness ratio has a greater impact on the initial stiffness of the members. The greater the width-to-thickness ratio of a member is, the greater the initial stiffness of the member.
(3) Based on the equilibrium condition, a practical formula considering the confinement coefficient for the ultimate bearing capacity of square CFST stub columns under axial loading with different material matches was proposed. The proposed formula shows a better calculation accuracy and clearer physical meaning of HSCFST compared with major code formulae.

## Figures and Tables

**Table 1:** Model parameter matching table.

*Types*	*f*_s_	*f*_c_	*L*	*B*	*D*	*t*	*D/t*	*L/D*
CS-CC	235	30	1500	500	500	3	167	500
CS-CC		60		500	500	6	83	
CS-CC		90		500	500	10	50	
CS-HC		120		500	500	3	167	
CS-HC		150		500	500	6	83	
CS-HC		180		500	500	10	50	
CS-CC	345	30	1500	500	500	3	167	500
CS-CC		60		500	500	6	83	
CS-CC		90		500	500	10	50	
CS-HC		120		500	500	3	167	
CS-HC		150		500	500	6	83	
CS-HC		180		500	500	10	50	
CS-CC	460	30	1500	500	500	3	167	500
CS-CC		60		500	500	6	83	
CS-CC		90		500	500	10	50	
CS-HC		120		500	500	3	167	
CS-HC		150		500	500	6	83	
CS-HC		180		500	500	10	50	
HS-CC	550	30	1500	500	500	3	167	500
HS-CC		60		500	500	6	83	
HS-CC		90		500	500	10	50	
HS-HC		120		500	500	3	167	
HS-HC		150		500	500	6	83	
HS-HC		180		500	500	10	50	
HS-CC	690	30	1500	500	500	3	167	500
HS-CC		60		500	500	6	83	
HS-CC		90		500	500	10	50	
HS-HC		120		500	500	3	167	
HS-HC		150		500	500	6	83	
HS-HC		180		500	500	10	50	
HS-CC	800	30	1500	500	500	3	167	500
HS-CC		60		500	500	6	83	
HS-CC		90		500	500	10	50	
HS-HC		120		500	500	3	167	
HS-HC		150		500	500	6	83	
HS-HC		180		500	500	10	50	
HS-CC	960	30	1500	500	500	3	167	500
HS-CC		60		500	500	6	83	
HS-CC		90		500	500	10	50	
HS-HC		120		500	500	3	167	
HS-HC		150		500	500	6	83	
HS-HC		180		500	500	10	50	

**Table 2:** Parameter value and comparison of steel constitutive model.

Match Type	Formula	*α*	*β*	*K*	Quantity	Equation (16)		FE	
						Average	Dispersion	Average	Dispersion
CS-CC	*N*_u_ = *f*_c_*A*_c_ + *Kf*_s_*A*_s_	0.96	0.19	1.20	74	1.02	0.083	1.08	0.043
HS-CC		0.87	0.21	1.14	68	0.96	0.083	0.98	0.062
HS-HC		0.95	0.10	1.07	20	1.03	0.080	1.02	0.061
CS-HC		0.97	0.07	1.06	19	1.00	0.091	0.99	0.058
total						1.00	0.065	1.02	0.038

**Table 3:** Summary of available formulas in well-known national codes.

Reference	Formulas	Average Values (*N*_u,exp_/*N*_u,ref_)					Dispersion Coefficient (*N*_u,exp_/*N*_u,ref_)				
		CS- CC	HS- CC	CS- HC	HS- HC	Total	CS- CC	HS- CC	CS- HC	HS- HC	Total
GB50936 (2014)	$\begin{array}{l} N_{0}=(1.212+B\theta +C\theta^{2})f_{c}A_{sc}\\ \theta =\frac{A_{s}}{A_{c}}\frac{f}{f_{c}} \end{array}$	1.06	1.17	0.91	1.84	1.17	0.300	0.473	0.296	0.733	0.552
EC4 (2004)	$\begin{array}{l} N_{EC4}=\eta_{a}A_{s}f_{y}+A_{c}f_{c}^{\prime }(1+\eta_{c}\frac{t}{D}\frac{f_{y}}{f_{c}^{\prime }})\\ \eta_{a}=0.25(3+2\bar{\lambda })\leq 1.0,\\ \eta_{c}=4.9-18.5\bar{\lambda }+17{\bar{\lambda }}^{2}\geq 1.0;\\ \bar{\lambda }=\sqrt{\frac{N_{pl,Rk}}{N_{cr}}},N_{pl,Rk}=A_{s}f_{y}+A_{c}f_{c}^{\prime }\\ N_{cr}=\frac{\pi^{2}(E_{s}I_{s}+0.6E_{c}I_{c})}{L^{2}} \end{array}$	0.99	1.00	0.93	1.03	0.99	0.131	0.125	0.139	0.104	0.172
BS 5400 (1979)	$N_{u}=A_{s}f_{y}/\gamma_{s}+0.675f_{cu}A_{c}/\gamma_{c}$	1.43	1.32	1.51	1.10	1.39	0.076	0.176	0.111	0.111	0.142
ACI-318 (2011)	$N_{ACI}=A_{s}f_{y}+0.85f_{c}^{\prime }A_{c}$	1.07	1.07	1.03	1.10	1.07	0.124	0.201	0.127	0.092	0.160

## Data Availability

Not applicable.

## Appendix A

**Table A1:** Comparison between finite element calculation results and literature experimental results of high-strength concrete-filled square steel tube stub columns.

Specimen Number	Literature	*D* × *t* × *L*/mm	*f*_cu_/MPa	*f*_y_/MPa	*N*_u,e_/kN	*N*_u,FE_/kN	*N*_u,Eq_/kN	*N*_u,e_/*N*_u,FE_	*N*_u,e_/*N*_u,Eq_
NS1	[35]	186×3×558	40	300	1555	1628	1694	0.96	0.92
NS7		246×3×738	47.5	300	3095	2918	3005	1.06	1.03
NS13		306×3×918	47.5	281	4003	4179	4281	0.96	0.93
NS16		306×3×918	58.75	281	4658	4985	5087	0.93	0.92
S2	[36]	127×4.34×609.6	32.5	357	1095	1144	1220	0.96	0.90
S3		127×4.55×609.6	29.75	322	1113	1189	1152	0.94	0.97
S4		127×5.67×609.6	29.75	312	1202	1225	1310	0.98	0.92
C10K6-1-6-1	[37]	150×4.5×1855	80.00	379.8	1895	1758	1841	1.08	1.03
C10K6-1-6-2		150×4.5×1855	80.00	379.8	1889	1758	1841	1.07	1.03
C10K6-1-6-3		150×4.5×1855	80.00	379.8	1886	1758	1841	1.07	1.02
C10K6-1-6-4		150×4.5×1855	80.00	379.8	1892	1758	1841	1.08	1.03
C10K6-1-6-5		150×4.5×1855	80.00	379.8	1862	1758	1841	1.06	1.01
C10K6-1-6-6		150×4.5×1855	80.00	379.8	1890	1758	1841	1.07	1.03
C12K6-1-6-1		150×4.5×1855	94.00	379.8	2066.1	1935	1984	1.07	1.04
C12K6-1-6-2		150×4.5×1855	94.00	379.8	2196.4	2017	2017	1.09	1.09
C12K6-1-6-3		150×4.5×1855	94.00	379.8	2096.1	1935	2182	1.08	0.96
C12K6-1-6-4		150×4.5×1855	94.00	379.8	2090.1	1935	2100	1.08	1.00
C12K6-1-6-5		150×4.5×1855	94.00	379.8	2006.7	1935	1853	1.04	1.08
C12K6-1-6-6		150×4.5×1855	94.00	379.8	2083.5	1935	1935	1.08	1.08
HSS1	[38]	110×5×330	35	750	1836	1819	2024	1.01	0.91
HSS2		110×5×330	35	750	1832	1866	2024	0.98	0.91
HSS8		160×5×480	37.5	750	2868	2918	3127	0.98	0.92
HSS9		160×5×480	37.5	750	2922	2964	3220	0.99	0.91
CSC40SD8	[39]	150×8.275×453	43.2	488.38	3500	3326	3188	1.05	1.10
CSC50SD9		150×8.275×451	55.3	488.38	3575	3518	3381	1.02	1.06
CR4-A-8	[40]	148×4.38×444	87	262	2108	2145	2211	0.98	0.95
CR4-A-2		148×4.38×444	35.4	262	1153	1135	1201	1.02	0.96
CR4-A-4-1		148×4.38×444	50.5	262	1414	1435	1501	0.99	0.94
CR4-A-4-2		148×4.38×444	50.5	262	1402	1435	1501	0.98	0.93
CR4-A-4-3		210×5.48×630	46	294	3183	2830	2961	1.10	1.07
CR4-C-4-3		210×4.5×630	46	277	2713	2534	2637	1.07	1.03
CR4-C-8		215×4.38×645	90.3	262	3837	4028	4221	0.92	0.91
CR4-D-4-1		323×4.38×645	51.375	262	4950	5311	5457	0.93	0.91
CR6-A-2		144×6.36×432	31.75	618	2572	2615	2831	0.98	0.91
CR6-A-4-1		144×6.36×432	50.625	618	2808	2866	3082	0.98	0.91
CR6-A-4-2		144×6.36×432	50.625	618	2765	2655	2871	1.04	0.96
CR6-A-8		144×6.36×432	87	618	3399	3125	3342	1.09	1.02
CR6-C-2		211×6.36×633	31.75	618	3920	5342	5663	1.08	1.02
CR6-D-4-1		319×6.36×957	51.375	618	7780	7138	7630	1.09	1.02
CR6-D-4-2		318×6.36×954	51.375	618	7473	7108	7598	1.05	0.98
CR6-D-8		319×6.36×957	95.1	618	10,357	10,568	11,060	0.98	0.94
CR8-A-2		120×6.47×360	31.75	835	2819	2794	3039	1.01	0.93
CR8-A-4-1		120×6.47×360	50.625	835	2957	2971	3216	1.00	0.92
CR8-A-4-2		120×6.47×360	50.625	835	2961	2971	3216	1.00	0.92
CR8-A-8		119×6.47×357	87	835	3318	3100	3343	1.07	0.99
CR8-C-2		175×6.47×525	31.75	835	4210	4343	4707	0.97	0.89
CR8-C-4-2		175×6.47×525	50.625	835	4542	4733	5097	0.96	0.89
CR8-C-8		175×6.47×525	87	835	5366	5121	5485	1.05	0.98
CR8-D-2		265×6.47×795	31.75	835	6546	6691	7249	0.98	0.90
CR8-D-4-1		264×6.47×792	51.375	835	7117	7286	7841	0.98	0.91
CR8-D-4-2		265×6.47×795	51.375	835	7172	7303	7860	0.98	0.91
CR8-D-8		265×6.47×795	90.3	835	8990	8613	9171	1.04	0.98
CR4-A-9		211×5.48×633	101.1	294	4773	4552	4950	0.99	0.96
CR4-C-9		211×4.5×633	101.1	277	4371	4282	4694	1.02	0.93
CR6-A-9		211×8.83×633	113.875	536	7008	7079	7008	0.99	0.95
CR6-C-9		204×5.95×612	101.1	540	5303	5766	5403	0.92	0.90
CR8-A-4-3		180×9.45×540	48.875	825	6803	6640	6587	1.02	1.03
CR8-A-9		180×9.45×540	101.1	825	7402	6786	7402	1.09	0.93
CR8-C-9		180×6.6×540	101.1	824	5873	5446	5873	1.08	0.91
R7-1	[41]	106×4×320	99	495	1749	1658	1739	1.05	1.01
R7-2		106×4×320	99	495	1824	1658	1739	1.10	1.05
R10-1		140×4×420	99	495	2752	2604	2712	1.06	1.01
R10-2		140×4×420	99	495	2828	2604	2712	1.09	1.04
R1-1		120×4×360	70	495	1701	1669	1760	1.02	0.97
R1-2		120×4×360	70	495	1657	1669	1760	0.99	0.94
R4-1		130×4×390	70	495	2020	1884	1984	1.07	1.02
R4-2		130×4×390	70	495	2018	1884	1984	1.07	1.02
R7-1		106×4×318	99	495	1749	1658	1739	1.05	1.01
R7-2		106×4×318	99	495	1824	1658	1739	1.10	1.05
R10-1		140×4×420	99	495	2752	2604	2712	1.06	1.01
R10-2		140×4×420	99	495	2828	2604	2712	1.09	1.04
A1	[42]	120×5.8×360	93	300	1697	1566	1773	1.08	0.96
A2		120×5.8×360	116	300	1919	1840	2078	1.04	0.92
A3-1		200×5.8×600	93	300	3996	3892	4243	1.03	0.94
A3-2		200×5.8×600	93	300	3862	3892	4162	0.99	0.93
A9-1		120×4×360	65	495	1739	1609	1701	1.08	1.02
A9-2		120×4×360	65	495	1718	1609	1701	1.07	1.01
A12-1		130×4×390	65	495	1963	1814	1914	1.08	1.03
A12-2		130×4×390	65	495	1988	1814	1914	1.10	1.04
HSSC1	[43]	110×5×330	64.50	701	2203.00	2048	2195	1.08	1.00
HSSC2		110×5×330	64.50	701	2234.00	2048	2195	1.09	1.02
HSSC3		140×5×420	64.50	701	2942.00	2842	3031	1.04	0.97
HSSC4		140×5×420	64.50	701	2840.00	2842	3031	1.00	0.94
80 × 80 × 4-C120-A		80×4×240	122.70	756	1504.00	1896	1898	1.00	1.00
80 × 80 × 4-C80-B		80×4×240	89.90	1022	1791	1687	1811	1.06	0.99
80 × 80 × 4-C120-B		80×4×240	122.70	1022	1898	1896	1898	1.00	1.00
100 × 100 × 4-C40-B		100×4×299.5	44.90	980	2009	1852	2003	1.08	1.00
100 × 100 × 4-C80-B		100×4×299.5	89.90	980	2177	2210	2360	0.99	0.92
100 × 100 × 4-C120-B		100×4×299.5	122.70	980	2266	2431	2266	0.93	0.89
120 × 120 × 4-C40-B		120×4×360	44.90	991	2557	2338	2522	1.09	1.01
120 × 120 × 4-C80-B		120×4×359	89.90	991	2853	2868	3052	0.99	0.93
120 × 120 × 4-C80-B-r		120×4×359	89.90	991	2798	2868	3052	0.98	0.92
120 × 120 × 4-C120-B		120×4×360	122.70	991	2950	3089	2950	0.95	0.88
SC-32-80	[30]	305×8.9×1200	120.00	560	14,116	14,401	14,116	0.98	0.93
SC-48-80		305×6.1×1200	120.00	660	12,307	12,749	12,307	0.97	0.86
SC-32-46		305×8.6×1200	120.00	259	11,390	10,942	11,999	1.04	0.95
SC-48-46		305×5.8×1200	120.00	471	11,568	11,138	11,568	1.04	0.91
S1	[44]	150×8×450	162.30	779	6536	6510	6536	1.00	0.99
S2		150×8×450	167.20	779	6715	6358	6715	1.06	1.00
S3		150×8×450	157.00	779	6616	6867	6616	0.96	1.02
S4		150×8×450	174.10	779	7276	7557	7276	0.96	1.06
S5		150×8×450	158.00	779	6974	6532	6974	1.07	1.07
S6		150×12×450	162.30	756	8585	7765	8585	1.10	1.08
S7		150×12×450	167.20	756	8452	7850	8452	1.08	1.06
S8		150×12×450	157.00	756	8687	7674	8687	1.03	1.10
S9		150×12×450	174.10	756	8730	8723	8730	1.00	1.07
S10		150×12×450	158.00	756	8912	8135	8912	1.10	1.00
S11		150×12.5×450	162.30	446	5953	6355	6049	0.94	0.98
S12		150×12.5×450	167.20	446	5911	6439	5703	0.92	1.04
S13		150×12.5×450	157.00	446	6039	6265	6572	0.96	0.92
S14		150×12.5×450	174.10	446	6409	6557	6557	0.98	0.98
S15		150×12.5×450	158.00	446	6285	6282	5976	1.00	1.05
C1-1	[45]	100.3×4.18×300	80.80	550	1490	1464	1552	1.02	0.96
C1-2		101.5×4.18×300	80.80	550	1535	1504	1593	1.02	0.96
C2-1		101.2×4.18×300	92.10	550	1740	1601	1691	1.09	1.03
C2-2		100.7×4.18×300	92.10	550	1775	1587	1676	1.12	1.06
C3		182.8×4.18×540	80.80	550	3590	3727	3890	0.96	0.92
C4		181.8×4.18×540	92.10	550	4210	4028	4191	1.05	1.00
C5-1		120.7×4.18×360	80.80	550	1450	1462	1550	0.99	0.94
C5-2		119.3×4.18×360	80.80	550	1425	1454	1543	0.98	0.92
C6-1		119.6×4.18×360	92.10	550	1560	1546	1635	1.01	0.95
C6-2		120.5×4.18×360	92.10	550	1700	1556	1645	1.09	1.03
C7-1		179.7×4.18×540	80.80	550	2530	2703	2838	0.94	0.89
C8-1		180.4×4.18×540	92.10	550	2970	2897	3031	1.03	0.98
C9-2		160.7×4.18×480	80.80	550	1820	1852	1959	0.98	0.93
C10-1		160.1×4.18×480	92.10	550	1880	1976	2083	0.95	0.90
C10-2		160.6×4.18×480	92.10	550	2100	1966	2073	1.07	1.01
C12-1		199.2×4.18×600	92.10	550	2900	2801	2936	1.04	0.99
C12-2		199.8×4.18×600	92.10	550	2800	2759	2893	1.01	0.97
